# Spectrum and frequencies of *BRCA1/2* mutations in Bulgarian high risk breast cancer patients

**DOI:** 10.1186/s12885-015-1516-2

**Published:** 2015-07-17

**Authors:** Rumyana Ivanova Dodova, Atanaska Velichkova Mitkova, Daniela Rosenova Dacheva, Lina Basam Hadjo, Alexandrina Ivanova Vlahova, Margarita Stoyanova Taushanova - Hadjieva, Spartak Stoyanov Valev, Marija Mitko Caulevska, Stanislava Dimitrova Popova, Ivan Emilov Popov, Tihomir Iliichev Dikov, Theophil Angelov Sedloev, Atanas Stefanov Ionkov, Konstanta Velinova Timcheva, Svetlana Liubomirova Christova, Ivo Marinov Kremensky, Vanio Ivanov Mitev, Radka Petrova Kaneva

**Affiliations:** 1 Molecular Medicine Center, Medical University of Sofia, 2 Zdrave str., 1431 Sofia, Bulgaria; 2 Department of Medical Chemistry and Biochemistry, Medical Faculty, Medical University of Sofia, 2 Zdrave str., 1431 Sofia, Bulgaria; 3 General and Clinical Pathology Clinic, University Hospital “Alexandrovska”, 1 Georgi Sofiiski str., 1431 Sofia, Bulgaria; 4 Department of General and Clinical Pathology, Medical University of Sofia, 1 Georgi Sofiiski str., 1431 Sofia, Bulgaria; 5 Clinic of Medical Oncology (Chemotherapy), Specialized Hospital for Active Treatment in Oncology, 6 “Plovdivsko pole” str., 1756 Sofia, Bulgaria; 6 Department of Surgery, University Hospital “Tsaritsa Yoana - ISUL”, 8 “Byalo more” str., 1527 Sofia, Bulgaria; 7 Medical Faculty, 8 “Byalo more” str., 1527 Sofia, Bulgaria; 8 Department of General and Liver-Pancreatic Surgery, University Hospital “Alexandrovska”, 1 Georgi Sofiiski str., 1431 Sofia, Bulgaria; 9 Medical Faculty, Medical University of Sofia, 1 Georgi Sofiiski str., 1431 Sofia, Bulgaria

**Keywords:** *BRCA1*, *BRCA2*, Breast cancer, Genetic testing, Mutations, Sequencing

## Abstract

**Background:**

About 3885 women are diagnosed with breast cancer and 1285 die from the disease each year in Bulgaria. However no genetic testing to identify the mutations in high-risk families has been provided so far.

**Methods:**

We evaluated 200 Bulgarian women with primary invasive breast cancer and with personal/ family history of breast cancer for the presence of unequivocally damaging germline mutations in *BRCA1/2* using Sanger sequencing.

**Results:**

Of the 200 patients, 39 (19.5 %) carried a disease predisposing mutation, including 28 (14 %) with a *BRCA1* mutation and 11 (5.5 %) with a *BRCA2* mutation. At *BRCA1*, 6 different mutations were identified, including 2 frameshifts, 1 nonsense and 1 missense that had been previously reported (c.5030_5033delCTAA, c.5263_5264insC, c.4603G > T, c.181 T > G), and 2 frameshifts, which were novel to this study (c.464delA, c.5397_5403delCCCTTGG). At *BRCA2*, 7 different frameshift mutations were identified, including 5 previously reported (5851_5854delAGTT, c.5946delT, c.5718_5719delCT, c.7910_7914delCCTTT,c.9098_9099insA) and 2 novel (c.8532_8533delAA, c.9682delA).

A *BRCA1* mutation was found in 18.4 % of women diagnosed with breast cancer at/or under the age of 40 compared to 11.2 % of women diagnosed at a later age; a *BRCA2* mutation was found in 4 % of women diagnosed at/or under the age of 40 compared to 6.5 % of women diagnosed at a later age. A mutation was present in 26.8 % patients with a positive family history and in 14.4 % of women with a negative family history.

The most prevalent mutation observed in 22 patients (11 %) was *BRCA1* c.5263_5264insC, a known Slavic mutation with founder effect in Eastern European and AJ communities. Other recurrent mutations were *BRCA2* c.9098-9099insA (2 %), *BRCA1* c.181T > G (1 %) and *BRCA2* c.5851_5854delAGTT (1 %). Notably, *BRCA1* c.5263_5264insC represented 56 % of all mutations identified in this series. Of the 22 patients with *BRCA1* c.5263_5264insC, 9 were diagnosed with early onset breast cancer, 11 with TNBCs, 4 with bilateral breast cancer, and 6 with both breast and ovarian cancer.

**Conclusions:**

This is the first comprehensive study of the *BRCA1/2* mutation spectrum in Bulgaria and will assist the establishment of efficient protocols for genetic testing and individualized risk assessment for Bulgarian breast/ovarian cancer patients and healthy individuals at a high-risk.

**Electronic supplementary material:**

The online version of this article (doi:10.1186/s12885-015-1516-2) contains supplementary material, which is available to authorized users.

## Background

Breast cancer (BC) is the second most common cancer in the world and, by far, the most frequent malignant disease among women with an estimated 1.67 million new cases diagnosed in 2012 (25 % of all cancer cases) [1]. Despite the advancement of diagnostic techniques and treatment in the last decade, BC is still the most frequent cause of cancer death in women in less developed regions (324.000 deaths, 14.3 % of total) and the second cause of cancer death in more developed regions (198.000 deaths, 15.4 %) after lung cancer [1].

The etiology of BC is multifactorial and includes both environmental and genetic factors, as well as genetic and epigenetic changes during progression. Up to 5–10 % of all BC cases and 10–15 % of all ovarian cancer (OC) cases are due to germline mutations in one of the two breast cancer susceptibility genes, *BRCA1* [MIM#113705] and *BRCA2* [MIM#600185] [2–4].

Germline mutations in *BRCA1* or *BRCA2* explain about 50 % of disease aggregation in severely affected BC and OC families while their prevalence is lower among BC and OC patients unselected for family history or age of diagnosis: *BRCA1* mutations are found in <1–7 % cases and *BRCA2* mutations in 1-3 % cases [5]. However, as high a proportion as 84 % has been proposed for certain families [6]. Higher prevalence is associated with a family history of BC and/or OC, early onset, male BC or multiple tumours such as bilateral breast cancer (BBC) or BC and OC in the same patient [5]. Furthermore, some histopathologic features such as lack of expression of estrogen, progesterone, and HER2 that define the biologically aggressive and difficult to treat Triple Negative Breast Cancers (TNBC) subtype, have been also attributed to deleterious changes mainly in *BRCA1* [7].

Pathogenic mutations in the *BRCA1* and *BRCA2* genes confer high risks of breast, ovarian, and contralateral BC. In a recent study the average cumulative risks by age 70 years for *BRCA1* carriers were estimated to be 60 % (95 % confidence interval [CI] = 44 to 75 %) for BC, 59 % (95 % CI = 43 to 76 %) for OC, and 83 % (95 % CI = 69 to 94 %) for contralateral BC [8]. For *BRCA2* carriers, the corresponding risks were 55 % (95 % CI = 41 to 70 %) for BC, 16.5 % (95 % CI = 7.5 to 34 %) for OC, and 62 % (95 % CI = 44 % to 79.5 %) for contralateral BC [8].

The prevalence of *BRCA1* and *BRCA2* mutations varies between ethnic groups and geographical areas [9]. At present, more than 1600 mutations in *BRCA1* and more than 1900 mutations in *BRCA2* have been described [10]. The spectrum of disease-associated *BRCA1* and *BRCA2* alleles includes frameshift, nonsense, and missense mutations altering protein function, splice mutations leading to truncation, as well as large genomic rearrangements [11, 12]. The majority of germline mutations identified in *BRCA1* and *BRCA2* are “private” or family-specific [12]. However, several examples of founder mutations have been described in certain geographical areas and ethnic communities where the *BRCA1* and *BRCA2* mutational spectra are limited to a few founders [13]. In fact, founder mutations have been described in Ashkenazi Jews (AJ), Icelandic and Finnish populations, in certain Dutch and French-Canadian communities, and in countries like Turkey, Pakistan, India, etc. [12–14].

Identification of *BRCA1/2* mutation carriers allows nondirective clinical decisions to be made in the management of high life time risk of BC/OC including follow-up, prophylactic mastectomy and salpingooophorectomy [7]. Furthermore, mutations in *BRCA1/2* have been shown to be predictive of good response to certain treatments, such as cisplatin and Poly (ADP)-Ribose Polymerase (PARP) inhibitors [7].

According to the National Cancer Registry, BC is the most common female malignancy in Bulgaria [15]. Around 3885 women are diagnosed with BC and 1285 die from the disease each year [15]. The first study of 20 Bulgarian familial BC patients was performed in 1998 aiming to develop a screening approach for the *BRCA1* gene [16]. No mutations but only benign polymorphisms were identified*,* most likely due to the small sample size, the low stringency of the selection criteria and the lack of complete analysis of *BRCA2*. Up to date no other surveys on *BRCA1*/*2* mutations in Bulgarian patients with familial BC/OC have been published.

The lack of sufficient genetic studies for inherited mutations in *BRCA1*/*2* genes in the Bulgarian population impedes the introduction of an effective mutation screening that would identify the individuals at high risk in BC/OC families. The aim of the present work was to conduct a genetic analysis for *BRCA1*/*2* germline mutations in a cohort of 200 Bulgarian women with BC, fulfilling the recognized international criteria [17, 18]. Direct sequencing of all coding exons and intron-exon junctions of both genes was performed. This is the first comprehensive study in Bulgaria aiming to ascertain the contribution of *BRCA1*/*2* germline mutations to familial BC in the Bulgarian population.

## Methods

### Participants

In the present study 200 unrelated female patients with primary BC, were selected from the Departments of Surgery and General and Clinical Pathology, University Hospital, “Alexandrovska”, Medical University of Sofia, and the Clinic of Medical Oncology, Specialized Hospital for Active Treatment in Oncology, Sofia, during their treatment and follow up procedures for the period of 2007–2012. All patients were of Bulgarian ethnicity, except one of Jewish origin. In personal interviews all clinical information, histopathology reports and family history were obtained from the patients. Their clinical features are summarized in Table 1. Sixty-one firstdegree relatives (8 diagnosed with BC/OC and 53 healthy) of 30 probands possessing a strong family history of BC/OC have been also recruited. They were considered for testing of possibly inherited damaging mutations. The cancer diagnosis of the affected relatives was verified by their personal clinical records.

**Table 1:** Clinical features of 200 Bulgarian patients with primary breast cancer selelected for age of diagnosis, family history and tumour characteristics

	N	Proportion of patients per feature (%)
Age at breast cancer diagnosis		
<40	76	38
41–60	99	49
>60	25	13
Bilateral breast cancer		
Yes	30	15
No	170	85
Personal history of ovarian cancer		
Yes	12	6
No	188	94
Family history of breast and ovarian cancer		
Cancer in 1^st^ or 2^nd^ relative		
Breast cancer dx < 60	73	36.5
Ovarian cancer, any age	11	5.5
Male breast cancer, any age		
Neither	104	52
Unknown	12	6
Triple negative (TNBC)		
Yes	28	14
No	172	86
Unknown		
Tumor hormone receptor status		
Estrogen receptor (ER)		
Positive	89	45
Negative	70	34
Unknown	41	21
Pogesteron receptor (PR)		
Positive	80	40
Negative	77	39
Unknown	43	21
Her2/neu		
Positive	82	41
Negative	60	30
Unknown	58	29
Stage		
1	48	24
2	103	51.5
3	15	7.5
4	1	0.5
Unknown	33	16.5
Grade		
I	6	3
II	70	35
III	34	17
Unknown	90	45
All patients	200	100 %

Probands were selected for *BRCA1/2* genetic testing according to their age of diagnosis, family history and tumour characteristics following the recognized Breast Cancer Linkage Consortium (BCLC) and National Comprehensive Cancer Network (NCCN) Criteria, summarized in Additional file 1: Table S1 [17, 18].

The distribution by criteria was as follows: 61 % (n = 122) fulfilled the BCLC criteria, 25 % (n = 50) - the NCCN criteria and 14 % (n = 28) were TNBC. The predominant was the group with early onset (38 %, n = 76), followed by the group with family history of BC (37 %, n = 73) in at least one first or second-degree relative diagnosed under the age of 60 (Table 1). Fifteen % (n = 30) of the patients were diagnosed with BBC, 6 % (n = 12) had a personal history of OC, whereas 5 % (n = 11) – family history of OC in at least one first or second-degree relative diagnosed at any age (Table 1). In addition to the TNBC group (14 %, n = 28) (Table 1), 14 % (24/172) of the patients selected by the BCLC and 2 NCCN criteria were also TNBC.

All deleterious mutations and some of the variants with unknown clinical significance (VUSs) found in the patients were validated in a control group of 96 healthy women matched by age and ethnicity to the patients, without family history of BC/OC. Only VUSs associated with BC in previous studies were screened in the control group.

The control group was collected through the clinical specialists participating in the study and included women undergoing regular prophylactic breast examinations in the University Hospital “Alexandrovska”, Medical University of Sofia, and Specialized Hospital for Active Treatment in Oncology, Sofia. They were defined as healthy based on the results of their last breast examinations signed by breast specialists and in addition were screened for lack of family history of BC/OC.

The study was approved by the Ethics Committee, Medical University of Sofia. All persons confirmed their agreement for participation upon signing an informed consent and donated 10 ml of blood for genetic analysis. The participants have also given consent for publishing their genetic results and relevant clinical data anonymously under a specific ID code assigned.

### Genetic analysis

Total genomic DNA was isolated from peripheral blood using a Chemagic Magnetic Separation Module and the Chemagic DNA Blood kits according to the manufacturer’s recommendations (Chemagen, Perkin Elmer).

In order to amplify all coding sequences and exon-intron junctions of the *BRCA1/2* genes, we have used a set of 81 primer pairs (33 for *BRCA1* and 48 for *BRCA2*) selected from the BIC database [10] or designed by ExonPrimer software [19] (Additional file 2: Table S2). PCR reactions were performed in 1X PCR buffer (Invitrogen), 0.4 mM of each dNTP, 1.5 mM MgCl_2_, 0.4 pmol of each primer and 1U of Taq DNA polymerase (Invitrogen) with 50 ng of genomic DNA in a final volume of 10 μl. Amplification conditions were identical for all amplicons of both genes, ranging in size from 190 to 720 bp, excluding the specific hybridization temperatures of the primer pairs given in Additional file 2: Table S2 initial denaturation at 94 °C (5 min); followed by 35 cycles of 94 °C (45 s), 51 °C - 63 °C (35 s), 72 °C (30 s) and 72 °C (10 min).

The mutation screening of *BRCA1/2* genes was performed using Sanger sequencing. PCR products were purified with ExoSapIT (Affymetrix) according to the manufacturer’s instructions – equal amounts of ExoSap and dH_2_O (0.4 μl each) were added to 1 μl of PCR product and incubated in a cycler for 30 min at 37 °C, followed by 15 min at 80 °C. Sequencing reactions were carried out with Big Dye® Terminator kit v3.1 (Life Technologies) in a final volume of 10 μl under the following conditions: 96 °C (5 min), 96 °C (20 s), 55 °C (20 s), 60 °C (2 min), 60 °C (5 min).

The fragments were analysed by automated Genetic Analyser ABI 3130xl (Applied Biosystems). Obtained sequences were examined for the presence of mutations by alignment with reference DNA sequence (GenBank U14680 for *BRCA1*; GenBank U43746 for *BRCA2*) using BLAST [20] and SeqScape TM v 2.0 (Applied Biosystems, USA) software packages. Any mutation found was confirmed in a second PCR reaction followed by sequencing in both forward and reverse directions.

### In silico analysis of sequence variants

Potential structural and functional effect of the missense VUSs was predicted by the following online tools: PolyPhen 2 (http://genetics.bwh.harvard.edu/pph/), SIFT (http://sift.jcvi.org/) and PROVEAN (http://provean.jcvi.org/index.php) [21–23].

## Results

In the current study 200 Bulgarian patients selected by the established genetic testing criteria were screened for mutations in the *BRCA1/2* genes by direct sequencing. The mean age of the patients at diagnosis was 49.5 (25–74) years. Thirty eight percent (n = 76) of the patients were under 40, 49.5 % (n = 99) between 41 and 60, and 12.5 % (n = 25) between 61 and 80 years of age (Table 1).

Upon direct sequencing, 13 different damaging mutations were identified, 6 in *BRCA1* (four frameshift, one nonsense, one missense) and 7 frameshift in *BRCA2* (Table 2). Of those 84.61 % (11/13) were frameshift mutations seen in 18 % (36/200) of the patients. None of the pathogenic mutations was found in the healthy controls.

**Table 2:** *BRCA1/2* damaging mutations in Bulgarian BC patients

Exon	HGVS nomenclature	BIC nomenclature	Protein nomenclature	Functional domain	Mutation type	Times observed
			BRCA1			
5	c.181 T > G	C61G	p.Cys61Gly	Ring finger	Missense	2
8	c.464delA	583delA	p.Gln155fs	-	Frameshift	1
15	c.4603G > T	E1535X	p.Glu1535Ter	AD1	Nonsense	1
17	c.5030_5033delCTAA	5149del4	p.Thr1677_Asn1678delinsIlefs	BRCT1/AD2	Frameshift	1
20	c.5263_5264insC	5382insC	p.Ser1755delinsSerProfs	linker	Frameshift	22
22	c.5397_5403delCCCTTGG	5515del7	p.Thr1799delins	BRCT2/AD2	Frameshift	1
			BRCA2			
11	c.5718_5719delCT	5946delCT	p.Asn1906_Ser1907 = fs	-	Frameshift	1
11	c.5851_5854delAGTT	6079del4	p.Ser1951_Leu1952delinsTrpfs	-	Frameshift	2
11	c.5946delT	6174delT	p.Ser1982Argfs	BRC repeat7	Frameshift	1
17	c.7910_7914delCCTTT	8138del5	p.Ala2637_Phe2638delinsAlafs	Helical Domain	Frameshift	1
20	c.8532_8533delAA	8760delAA	p.Glu2844fs	Tower	Frameshift	1
23	c.9098_9099insA	9326insA	p.Thr3033delinsThrSerfs	OB2	Frameshift	4
27	c.9682delA	9908delA	p.Gln3227fs	-	Frameshift	1

### BRCA1 mutations

In the *BRCA1* gene we have identified six unequivocally deleterious mutations of which four frameshift, one nonsense and one missense (Table 2). Among the frameshift mutations two: c.5030_5033delCTAA and c.5263_5264insC with frequencies of 0.5 % and 11 %, respectively, had been previously reported and two, namely c.464delA and c.5397_5403delCCCTTGG with frequencies of 0.5 % each were novel (Table 2). The mutation c.464delA, located in exon 8, was detected in a patient BC134 diagnosed with TNBC at the age of 54 with three cases of BC in her pedigree (Table 3). The second novel mutation c.5397_5403delCCCTTGG in exon 22 was also found in an individual with TNBC (BC205) diagnosed at the age of 51 with family history of BC and PC (Table 3).

**Table 3:** Clinical data of the carriers of damaging *BRCA1* mutation

BC№	HGVS nomenclature	Diagnosis and age of onset	ER	PR	HER2	TNM	Grade	Stage	Other cancer in patient (age of onset)	Family history of cancer and age of diagnosis
3	c.5263_5264insC	BC (36)	NA	NA	NA	NA	NA	NA	DC (41)	Grandmother - BC (NA)
6	c.5263_5264insC	IDC (43)	-	1+	NA	pT2N0Mx	NA	II	OC (45)	Mother – BC (50), sister - BBC (42/53), grandmother – BC and OC (50)
7	c.5263_5264insC	BBC (42)	3+/−	3+/−	1+/1+	pT1N0M0/pT1bpN1M0	G2/G2	I/IIB		Grandmother - BC (76), aunt BC(81), father seminoma (NA), cousin with cancer of the oral cavity (NA)
17	c.5263_5264insC	IDC (34)	2+	2+	NA	NA	G2	NA		No
21	c.5263_5264insC	IDC (48)	NA	NA	NA	NA	NA	NA	OC (48)	Grandfather - GC (70), grandfather – BT (NA), PC (NA), aunt - CGO (50), cousin - BC (54)
28	c.181 T > G	BBC (52/60)	NA	NA	NA	NA	NA	NA	OC (52)	Brother with bone cancer (47)
37	c.4603G > T	IDC (37)	-	-	-	pT1cN1M0	G2	II	FtC (41)	Grandfather - PC (76), grandfather - LC (69), grandmother - CRC (60)
39	c.5263_5264insC	BBC(37/55)	NA/-	NA/-	NA/-	NA	NA	NA		Uncle - PrC (66)
73	c.5263_5264insC	IDLC (53)	-	-	-	pT2pN0M0	G3	II		Mother - BC (47) and CRC (71)
79	c.5263_5264insC	IDC (42)	NA	NA	1+	NA	NA	NA		Grandmother - GC(70), mother – BBC (27/30), uncle – leukemia (67), grandmother - CLv (75)
99	c.5263_5264insC	IDC (40)	NA	NA	NA	pT1N0M0	G2	NA	OC (46)	Grandmother – unknown cancer (>80), mother-CrC (NA), mother’s sister – Paget’s disease of the nipple (NA)
111	c.5263_5264insC	ACC (36)	-	-	-	NA	G1	NA		No
121	c.5263_5264insC	IDC (34)	-	-	-	pT1N0M0	G3	I		Father – pelvic cancer (NA), cousin - BC (30)
132	c.181 T > G	IDC (39)	-	-	-	pT1pN0M0	G2	I		Grandmother - BC (60), mother -BBC (32/40)
134	c.464delA	IDC (54)	-	-	-	pT1N1M0	G3	II		Aunt - BC (47), grandmother and grandmother’s sister - BC(NA), father - LC (NA)
140	c.5263_5264insC	IDC (32)	1+	2+	2+	pT2pN0M0	G2	IIa		Mother - BBC (31/37), grandmother - EC, CRC (50/53), grandfather - LC (59), grandmother - BC (NA)
142	c.5263_5264insC	IDC (31)	-	-	-	pT1pN0M0	G2-3	I		No
143	c.5263_5264insC	IDC (51)	-	-	-	pT2N0M0	G3	I	OC (51)	Uncle - PC (68), grandmother LC (44), cousin - OC (37)
152	c.5263_5264insC	BBC (41)	NA	NA	NA	NA	NA	NA	OC (58)	Mother - BBC (NA)
155	c.5263_5264insC	BC (29)	-	-	-	NA	NA	NA		Grandmother - BC, ThC (50/70), cousin - BC (50), cousin - OC (48)
161	c.5263_5264insC	IDC (43)	-	-	-	NA	NA	NA		Mother – OC (62)
164	c.5263_5264insC	IDC (45)	-	-	-	NA	NA	NA		Mother - BC (NA)
171	c.5263_5264insC	BC (53)	-	-	-	NA	NA	NA	OC (NA)	Grandmother - BC (43), mother - BC (53) and OC (63)
175	c.5263_5264insC	MBC (33)	-	-	-	pT1сN2M0	G2	II		No
194	c.5030_5033	IDC (63)	-	-	-	pT1N0M0	G2	NA		No
	delCTAA									
190	c.5263_5264insC	IDC (33)	-	-	-	pT1bpN0M0	G2	I		Grandmother - BC (47), mother - OC (52), grandfather - CRC (62)
204	c.5263_5264insC	BBC (35/48)	NA	NA	NA	NA	NA	NA		Mother with bladder cancer in doubt
205	c.5397_5403	IDC (51)	-	-	-	pT1pN1M0	G3	II		Grandmother - BC (30), father – PC (NA)
	delCCCTTGG									

BC№ - case identifier, BC – breast cancer, BBC – bilateral breast cancer, IDC – invasive ductal carcinoma, ILC - invasive lobular carcinoma, IDLC - infiltrating ductal/ lobular cancer, MBC - medullary breast cancer, ACC – adenoid cystic carcinoma, ThC – throat cancer, LyC – laryngeal cancer, PC – prostate cancer, LC – lung cancer, CRC – colorectal carcinoma, PrC – pancreatic cancer, CLv – cancer of the liver, GC - gastric cancer, OC - ovarian cancer, BT - brain tumor, EC - endometrial cancer, CGO – cancer of genital organs, DC – dysplasia of the cervix, FtC – fallopian tube cancer, NA – not available, “-“negative for ER, PR and HER2

The deletion c.5030_5033delCTAA in *BRCA1* exon 17 was observed in one patient (BC194) without family history diagnosed with TNBC at the age of 63 (Table 3). Twenty-two of the patients (11 %) harboured the mutation c.5263_5264insC in *BRCA1* exon 20 (Table 3). Of those six (BC6, BC21, BC99, BC143, BC152 and BC171) had developed both BC and OC, four were diagnosed with BBC (BC7, BC39, BC152 and BC204). Eleven of the cases were TNBC (BC73, BC111, BC121, BC142, BC143, BC155, BC161, BC164, BC171, BC175 and BC190) of which 5 (BC111, BC121, BC142, BC155 and BC190) developed the disease before the age of 40. Altogether in 9 of the patients, the c.5263_5264insC mutation correlated with early onset (BC3, BC39, BC140, BC111, BC121, BC142, BC155, BC190 and BC204).

In addition to the listed above indels we found two deleterious point mutations that had previously been reported (Table 2): one nonsense (c.4603G > T) with a frequency of 0.5 % and one missense (c.181T > G) with a frequency of 1 %. The mutation c.4603G > T was carried by a patient BC37 with early onset (Table 3) while c.181T > G was detected in two patients (BC28 and BC132), with early onset and BBC, respectively (Table 3).

### BRCA2 mutations

Seven damaging frameshift mutations were found in *BRCA2* (Table 2), of which five had previously been reported: c.5851_5854delAGTT, c.5946delT, c.5718_5719delCT, c.7910_7914delCCTTT, c.9098_9099insA; and two novel: c.8532_8533delAA and c.9682delA. The most frequent *BRCA2* frameshift mutation c.9098_9099insA, located in exon 23, was observed in four patients (BC32, BC81, BC85 and BC88) with familial BC (2 %). In addition one of them (BC88) had TNBC (Table 4).

**Table 4:** Clinical data of the carriers of damaging *BRCA2* mutation carriers

BC№	HGVSnomenclature	Diagnosis and age of onset	ER	PR	HER2	TNM	Grade	Stage	Other cancer in patient	Family history of cancer and age of diagnosis
19	c.7910_7914	BBC (31/37)	NA/ NA	NA/ NA	NA/ NA	NA/ NA	NA/ NA	NA		Cousin - BBC (41/49), father - GC (NA), aunt - OC (NA)
	delCCTTT									
24	c.9682delA	BBC (52)	NA	NA	NA	NA	NA	NA		NA
32	c.9098_9099insA	IDC (50)	-	-	3+	NA	G3	NA		Aunt – BC (NA)
52	c.5946delT	BBC (41/68)	NA/	NA/	NA/ NA	NA/ NA	NA/ NA	NA	OC (59)	Mother - BC (36) sister - BC (46) and CRC (65), father - LC, ethnicity - Jewish
			3+	3+						
58	c.5851_5854	IDC (48)	3+	3+	2+	pT1N0Mx	G3	NA		Mother – BC (NA), brother and sister of the mother – CRC (NA), her father – LC (NA), cousin – CRC (NA), a brother of the father – CLv (NA) and his children - BC (35) and - LC (50)
	delAGTT									
76	c.5851_5854	IDLC (53)	-	-	-	pT1pN0M0	G2	NA		Cousin - BC (65), father - StC (54)
	delAGTT									
81	c.9098_9099insA	BC (59)	NA	NA	NA	NA	NA	NA		Sister - BC (52), aunt - BC (67)
85	c.9098_9099insA	IDC (52)	3+	-	-	pT1pN2pMx	G3	III		Aunt - BC (42), grandfather - head and neck cancer (NA)
87	c.8532_8533	BBC (30/37)	3+/	-	NA	pT1bpN0M0/	G2/	I/I		Mother BBC (NA)
	delAA		3+	/3+	/1+	pT1bpN0M0	NA			
88	c.9098_9099insA	IDLC (61)	-	-	-	pT2pN0M0	G2-3	Iia		Grandfather - ThC (48), mother - BC (60), two cousins - BC (34/47)
90	c.5718_5719	IDC (35)	2+	3+	3+	pT2pN2M0	G2	III		Grandfather - LyC (NA), grandmother - unclear malignant disease, most likely melanoma (NA)
	delCT									

BC№ - case identifier, BC – breast cancer, BBC – bilateral breast cancer, IDC – invasive ductal carcinoma, ILC - invasive lobular carcinoma, IDLC - infiltrating ductal/ lobular cancer, MBC - medullary breast cancer, ACC – adenoid cystic carcinoma, ThC – throat cancer, LyC – laryngeal cancer, PC – prostate cancer, LC – lung cancer, CRC – colorectal carcinoma, PrC – pancreatic cancer, CLv – cancer of the liver, GC - gastric cancer, OC - ovarian cancer, BT - brain tumor, EC - endometrial cancer, CGO – cancer of genital organs, DC – dysplasia of the cervix, FtC – fallopian tube cancer, NA – not available, “-“negative for ER, PR and HER2

The second in frequency *BRCA2* frameshift mutation c.5851_5854delAGTT, located in exon 11, was found in two patients (1 %): BC76 with a family history of BC and stomach cancer, diagnosed with TNBC at the age of 53, and BC58 with family history of BC and CRC, diagnosed with BC at the age of 48 (Table 4).

The rest of the *BRCA2* frameshift mutations were seen only once in our study with a frequency of 0.5 % each (Table 2). Two deletions in exon 11: c.5718_5719delCT and c.5946delT were observed respectively in a patient BC90 with early onset (35 years) and in a Jewish patient BC52 with family history of BC, diagnosed with both BBC (at the age of 41/68) and OC (at the age of 59) (Table 4).

The deletion c.7910_7914delCCTTT, located in exon 17, was present in a patient BC19 with early onset, BBC (at the age of 37/41) and family history of BC/OC (Table 4).

One of the novel *BRCA2* deletions c.8532_8533delAA, located in exon 20, was found in a patient BC87 with BBC and early onset (at the age of 30/37). Interestingly, the second new *BRCA2* deletion c.9682delA, located in exon 27, was also observed in a patient (BC228) with BBC (Table 4).

### Unclassified variants

Additional 50 sequence variants were identified at *BRCA1* and *BRCA2*, with either benign or unknown pathogenic effect. All of them were named sequence variants due to inconsistency in their classification in the databases. The spectrum of unclassified variants included missense, synonymous and intronic variants, as well as one inframe deletion (Table 5).

**Table 5:** Unclassified *BRCA1/2* variants in Bulgarian BC patients

Exon	HGVS nomenclature	BIC nomenclature	Protein nomenclature	Functional domain	Mutation type	MAF in Bulgarian patients	Analysed patients (n)	MAF in healthy controls	Analysed healthy controls (n)	MAF in Europeans
BRCA1										
5	c.139 T > G	C47G	p.Cys47Gly	ring finger	missense	0.002	200	0.000	96	NA
8	c.536A > G	Y179C	p.Tyr179Cys	-	missense	0.002	194	0.000	96	0.001
11	c.736 T > G	L246V	p.Leu246Val	-	missense	0.002	200	-	-	0.002
11	c. 1067A > G	Q356R	p. Gln356Arg	-	missense	0.072	194	0.052	96	0.049
11	c.1456 T > C	F486L	p.Phe486Leu	-	missense	0.002	200	-	-	0.001
11	c.1648A > C	N550H	p.Asn550His	-	missense	0.002	200	0.00	96	0.001
11	c.2077G > A	D693H	p.Asp693Asn	-	missense	0.065	192	0.083	96	0.097
11	c.2612C > T	P871L	p.Pro871Leu	-	missense	0.350	193	0.391	96	0.336
11	c.3113A > G	E1038G	p.Glu1038Gly	-	missense	0.335	195	0.349	96	0.358
11	c.3119G > A	S1040N	p.Ser1040Asn	-	missense	0.023	195	0.026	96	0.580
11	c.3548A > G	K1183R	p.Lys1183Arg	-	missense	0.339	193	0.302	96	0.332
11	c.3999 T > C	V1333A	p. Val1333Ala	AD1	missense	0.002	200	0.00	96	-
16	c.4837A > G	S1613C	p.Ser1613Gly	AD2	missense	0.323	193	0.318	96	0.314
16	c.4956G > A	M1652I	p.Met1652Ile	BRCT1/AD2	missense	0.036	193	0.016	96	0.005
9	c.591C > T	C197C	p.Cys197=	-	cds-synon	0.007	193	0.00	96	0.001
11	c.2082C > T	S694S	p.Ser694=	-	cds-synon	0.323	192	0.318	96	0.280
11	c.2311 T > C	L771L	p.Leu771=	-	cds-synon	0.233	191	-	-	0.332
13	c.4308 T > C	S1436S	p.Ser1436=	AD1	cds-synon	0.337	194	-	-	0.332
7	c.441 + 36_441 + 38delCTT	IVS7 + 36delCTT	-	-	IVS	0.194	195	0.354	96	NA
8	c.442-34C > T	IVS7-34 C > T	-	-	IVS	0.230	194	0.224	96	0.257
9	c.548-58delT	IVS8-58delT	-	-	IVS	0.196	193	0.385	96	0.331
15	c.4485-63C > G	IVS14-63C > G	-	-	IVS	0.330	197	-	-	0.321
18	c.5075-53C > T	IVS17-53 C > T	-	-	IVS	0.010	194	-	-	0.034
20	c.5277 + 56delGTA	IVS20 + 56insGTA	-	-	IVS	0.002	200	0.00	96	-
BRCA2										
10	c.865A > C	N289H	p.Asn289His	-	missense	0.050	185	-	-	0.031
10	c.978C > A	S326R	p.Ser326Arg	-	missense	0.010	185	-	-	0.001
10	c.1114A > C	H372N	p.Asn372His	-	missense	0.283	185	-	-	0.300
11	c.2971A > G	N991D	p.Asn991Asp	-	missense	0.050	185	-	-	0.031
11	c.3515C > T	S1172L	p.Ser1172Leu	-	missense	0.002	185	0.00	96	0.001
11	c.5744C > T	T1915M	p.Thr1915Met	-	missense	0.017	200	0.016	96	0.032
11	c.6100C > T	R2034C	p.Arg2034Cys	-	missense	0.002	200	-	-	0.018
11	c.6322C > T	R2108C	p.Arg2108Cys	-	missense	0.002	200	-	-	0.001
15	c.7469 T > C	I2490T	p.Ile2490Thr	Helical Domain	missense	0.002	185	-	-	0.000
23	c.9104A > C	Y3035S	p.Tyr3035Cys	OB2	missense	0.005	200	0.005	96	NA
11	c.4146_4148delAGA	E1382del	delinsAsp	-	Cds_indel	0.002	200	-	-	NA
10	c.1365A > G	S455S	p.Ser455=	-	Cds-synon	0.030	185	-	-	0.031
11	c.2229 T > C	H743H	p.His743=	-	Cds-synon	0.035	184	-	-	0.031
11	c.3396A > C	K1132K	p.Lys1132=	-	Cds-synon	0.285	182	-	-	0.250
11	c.3807 T > C	V1269V	p.Val1269=	-	Cds-synon	0.189	185	-	-	0.226
14	c.7242A > G	S2414S	p.Ser2414=	-	Cds-synon	0.184	184	-	-	0.190
2	c.-26G > A	203G > A	-	-	UTR-5	0.247	190	-	-	0.199
27	c.*105A > C	IVS27 + 104A > C	-	-	UTR-3	0.183	185	-	-	0.274
4	c.425 + 67A > C	IVS4 + 67A > C	-	-	IVS	0.050	184	-	-	0.031
4	c.425 + 147G > T	IVS4 + 147G > T	-	-	IVS	0.040	184	-	-	0.062
5_6	c.426-89 T > C	IVS4-89 T > C	-	-	IVS	0.040	184	-	-	0.031
8	c.681 + 56C > T	IVS8 + 56C > T	-	-	IVS	0.060	185	-	-	0.133
11	c.6841 + 80_6841 + 83	IVS11 + 80	-	-	IVS	0.289	185	-	-	0.294
	delTTAA	delTTAA								
13	c.7007 + 134_7007 + 135 insTTATAAAAT	IVS13 + 164 delTTATAAAAT	-	-	IVS	0.032	186	-	-	0.043
15	c.7617 + 28C > A	IVS15 + 28 C > A	-	-	IVS	0.002	200	-	-	-
17	c.7806-14 T > C	IVS16-14 T > C	-	-	IVS	0.320	187	0.469	96	0.465

In total 24 missense VUSs were identified, of which 14 in *BRCA1*and 10 in the *BRCA2* (Table 5). According to their minor allele frequencies (MAFs) in the patients they can be divided into two groups: rare variants with MAFs between 0.0025 and 0.005, and frequent variants with MAFs > 0.005. We have compared the MAFs of the unclassified missense variants found among patients with other Europeans [24] and with healthy controls (only for the variants associated with BC in previous studies). The studied variants in the control group included VUSs in exons 5 (c.139 T > G); 7 (c.441 + 36_441 + 38delCTT); 8 (c.536A > G, c.442-34C > T); 9 (c.591C > T, c.548-58delT); 11 (c.1067A > G, c.1648A > C, c.2077G > A, c.2612C > T, c.3113A > G, c.3119G > A, c.3548A > G, c.3999 T > C, c.2082C > T); 16 (c.4837A > G, c.4956G > A); and 20 (c.5277 + 56delGTA) of *BRCA1* and the variants in exons 11 (c.3515C > T, c.5744C > T); 17 (c.7806-14 T > C); and 23 (c.9104A > C) of *BRCA2* (Table 5). Variants for which no information for association with BC was found in the databases were not screened in the healthy controls but were included in Table 5. In addition *in silico* analysis was conducted in order to predict potential deleterious effect of all detected missense VUSs (Table 6).

**Table 6:** Assessment of the clinical effect of unclassified *BRCA1/2* missense variants detected in Bulgarian BC patients

Exon	HGVS nomenclature	BIC nomenclature	BIC	UMD	POLYPHEN2	SIFT	PROVEAN
BRCA1							
5	c.139 T > G	C47G	unknown	-	probably damaging	damaging	neutral
8	c.536A > G	Y179C	unknown	neutral	damaging	tolerated	neutral
11	c.736 T > G	L246V	unknown	neutral	probably damaging	tolerated	neutral
11	c. 1067A > G	Q356R	unknown	neutral	probably damaging	damaging	deleterious
11	c.1456 T > C	F486L	unknown	neutral	benign	tolerated	neutral
11	c.1648A > C	N550H	unknown	neutral	probably damaging	damaging	deleterious
11	c.2077G > A	D693H	no	neutral	benign	tolerated	deleterious
11	c.2612C > T	P871L	no	neutral	benign	tolerated	neutral
11	c.3113A > G	E1038G	no	neutral	possibly damaging	damaging	neutral
11	c.3119G > A	S1040N	unknown	neutral	probably damaging	damaging	neutral
11	c.3548A > G	K1183R	no	neutral	benign	tolerated	neutral
11	c. 3999 T > C	V1333A	-	-	possibly damaging	tolerated	neutral
16	c.4837A > G	S1613C	no	neutral	benign	damaging	neutral
16	c.4956G > A	M1652I	unknown	neutral	benign	tolerated	neutral
BRCA2							
10	c.865A > C	N289H	no	neutral	benign	tolerated	neutral
10	c.978C > A	S326R	no	neutral	benign	tolerated	neutral
10	c.1114A > C	H372N	no	neutral	benign	tolerated	neutral
11	c.2971A > G	N991D	no	polymorphism	benign	tolerated	neutral
11	c.3515C > T	S1172L	unknown	likely neutral	probably damaging	damaging	deleterious
11	c.5744C > T	T1915M	no	neutral	benign	tolerated	neutral
11	c.6100C > T	R2034C	unknown	neutral	probably damaging	tolerated	deleterious
11	c.6322C > T	R2108C	unknown	UV	benign	tolerated	neutral
15	c.7469 T > C	I2490T	no	UV	benign	tolerated	neutral
23	c.9104A > C	Y3035S	unknown	UV	benign	damaging	Neutral

Six rare *BRCA1* missense variants were seen in patients (Table 5): c.139 T > G, c.536A > G, c.736 T > G, c.1456 T > C, c.1648A > C and c.3999 T > C. Of those c.3999 T > C, causing a replacement of alanine to valine at codon1333 has never been reported. Even though c.3999 T > C was observed in a patient BC20 with BBC and early onset in the absence of other pathogenic mutations, the *in silico* analysis did not confirm its pathogenic effect (Table 6).

The missense variants c.139T > G had been predicted to be deleterious in previous functional studies [25]. Inour study it was found in one patient (0.5 %) with TNBC BC10 and appeared to be clinically important according to wo prediction programs (POLYPHEN 2 and SIFT), while PROVEAN predicted it as neutral (Table 6).

One patient with family history of BC117 was the only carrier of three rare missense variants c.536A > G, c.1456T > C and c.1648A > C, which were not seen in the control group (Table 5). Another rare missense *BRCA1* variant c.736T > G, listed as variant of unknown significance (VUS) in BIC [10] and neutral in UMD (Universal Mutation Database) [26], was found in patient BC30 with BBC (Table 5).

Eight missense variants were observed in *BRCA1* with MAF>0.005 in both patients and controls (Table 5). They were designated as VUS in BIC and neutral in UMD databases (Table 5). Only two of them (c.1067A > G and c.3113A > G) appeared to be deleterious according to the conducted *in silico* analysis (Table 6).

Four synonymous and 6 intronic variants were also detected in the *BRCA1* gene (Table 2). Of those c.5277 + 62_c.5277 + 64delGTA had not previously been reported and was seen in two patients (1 %) but not in the control group (Table 5). The variant c.591C > T was more frequent in patients (MAF = 0.007), but was not found in the control group and was rare in other Europeans (MAF = 0.001) (Table 5). All other variants had MAF > 0.005 in both patients and other Europeans, except c.441 + 36_441 + 38delCTT, for which data about MAF were not available in the databases (Table 2). Variants c.2082C > T, c.441 + 36_441 + 38delCTT, c.442-34C > T and c.548-58delT were also genotyped and found with high frequencies (MAF > 0.005) in the healthy controls (Table 5).

Five rare missense variants (MAF < 0.005) were identified in *BRCA2* (Table 5). Three of them c.6322C > T, c.7469 T > C andc.9104A > C were classified as VUS in both BIC [10] and UMD [26] databases, while c.3515C > T and c.6101G > A were designated as VUS only in BIC (Table 5). In UMD c.3515C > T was suggested to be likely neutral but no information was available for c.6101G >A (Table 5). The *in silico* analysis predicted pathogenic effect of both c.3515C > T and c.6101G > A (Table 6).

We found four frequent (MAF > 0.005) missense variants in *BRCA2* (Table 5). None of them was predicted to be pathogenic upon *in silico* analysis (Table 6). In addition we detected one in frame deletion of glutamate in exon 11- c.4146_4148delAGA (MAF = 0.0025), which was classified as VUS in both BIC [10] and LOVD (Leiden Open Variation Database) [27].

The other *BRCA2* sequence variants were observed with high frequencies in patients (MAF > 0.005), similar to those found in other Europeans (Table 5). Among them seven were synonymous replacements; two variants –c.-26G > A and c.*105A > C, were localized in 5′-UTR- and 3′-UTR, respectively and 8 were intronic. One of the intronic variants c.7617 + 28C > A was novel (Table 5).

## Discussion

This is the first comprehensive study aiming to ascertain the contribution of *BRCA1/2* germline mutations to BC development in the Bulgarian population, where 1285 women die from the disease each year [15]. The thorough mutation screening of all coding sequences and exon-intron junctions of *BRCA1/2* genes in a cohort of 200 Bulgarian women with primary invasive BC and with personal or family history of BC/OC, selected according to the recognised international criteria [17, 18] led to the identification of pathogenic mutations in 19.5 % of them (39/200).

We have found 13 unequivocally deleterious mutations of which 6 in *BRCA1* and 7 in *BRCA2* gene (Table 2). The predominant mutations seen in 18 % of the studied group of patients were frameshift mutations leading to truncated proteins with impaired function. In total 11 (84.6 %, 11/13) indels were seen, 4 in *BRCA1* and 7 in *BRCA2* The other two mutations identified were nonsense and missense mutation in *BRCA1*, each accounting for 7.7 % (1/13) of all disease causing mutations.

The median age of diagnosis was 46 (range 29–63 years) in the 28 BC with a *BRCA1* mutation (Table 3) and 45.5 (range 30–61 years) in the 11 BC with a *BRCA2* mutation (Table 4), compared with a median age of diagnosis of 49.6 years (range 25–74 years) for the 161 cases without a mutation. A *BRCA1* mutation was found in 18.4 % (14 of 76) women diagnosed with BC at or under the age of 40 compared to 11.2 % (14 of 124) of women diagnosed at a later age (Table 3); a *BRCA2* mutation was found in 4 % (3 of 76) women diagnosed with BC at or under the age of 40 compared to 6.5 % (8 of 124) of women diagnosed at a later age (Table 4). A mutation was present in 26.8 % (22 of 82) BC patients with a positive family history and in 14.4 % (17 of 118) of women with a negative family history.

### BRCA1 mutations

The most prevalent mutation observed in 22 patients (11 %) was *BRCA1* c.5263_5264insC, a known Slavic mutation with founder effect in Eastern European as well as AJ communities, (Table 2). This frameshift mutation is the second most frequently reported in BIC database [10]. It has been found with various frequencies in high risk BC/OC families from Poland (34 %), Russia (14 %), Hungary (14 %), Slovenia (13 %), AJ (10 %), Greece (8 %), Germany (4 %), and Italy (3 %) [13]. The c.5263_5264insC was not found in Spain and Portugal and it has been observed with a low frequency in the Netherlands, Belgium and Scandinavian countries [13]. In Russia, Belarus, Poland, Latvia, Czech Republic, Greece and Lithuania it accounts for respectively 94 %, 73 %, 60 %, 55 %, 37–52 %, 46 % and 34 % of all *BRCA1* mutations [13].

Being a founder mutation in Greece [28], c.5263_5264insC has been detected in other Balkan countries such as Romania, Turkey, Serbia, Croatia, and Macedonia but without a founder effect [29–33]. Apparently Bulgaria is the second Balkan country in which the most prevalent Slavic mutation c.5263_5264insC has a founder effect accounting for a very high proportion, about 76 % (22/29) from all detected *BRCA1* mutations (Table 3), with high intermediate frequency (11 %) compared to the other Central and Eastern European countries [13].

Originally c.5263_5264insC was described as a founder mutation in AJ, but recent haplotype analysis suggested that it most likely originated in Northern Russia or possibly Scandinavia, between 1800 and 1500 years ago, and was subsequently spread to the various populations from East to West and nearly worldwide [34]. A common ancestor for families with c.5263_5264insC mutation, reported from Europe, Brazil, North America and India is evident [34–36].

We suppose that in Bulgaria the mutation c.5263_5264insC also has a Slavic origin since the modern Bulgarian nation originated as a mixture of Slavic, Thracian and Bulgar tribes more than 1300 years ago. A detailed haplotype analysis is necessary to prove this hypothesis.

The increased risk of BC and OC in c.5263_5264insC mutation carriers has been estimated at 67 % (95 % CI, 36–83 %) and 33 % (95 % CI, 8–50 %), respectively [5]. At a molecular level it leads to a stop codon at position 1829 and respectively to a truncated protein that lacks its C-terminal BRCT motif. In our study the presence of the c.5263_5264insC mutation in the patients was associated with early onset in 9 patients (BC3, BC39, BC140, BC111, BC121, BC142, BC155, BC190 and BC204), BBC in 4 patients (BC7, BC39, BC152 and BC204), two subsequent events of BC and OC in 6 patients (BC6, BC21, BC99, BC143, BC152 and BC171) and/or TNBC in 11 patients (BC73, BC111, BC121, BC142, BC143, BC155, BC161, BC164, BC171, BC175 and BC190) (Table 3). Several carriers were also identified among the relatives of the patients with c.5263_5264insC (one individual with OC, another with both BC and OC, as well as three healthy individuals).

The mutation c.5030_5033delCTAA located in *BRCA1* exon 17 is a prevalent frameshift mutation reported in at least five French families with BC/OC and most likely originating from a common ancestor (13). It has been also detected in BC patients with early onset from USA (0.35 %, 1/282) and Taiwan (2.8 %, 1/36) [37, 38]. We observed the mutation in one patient (0.5 %, 1/200) with TNBC (BC194) diagnosed at the age of 63 (Table 4).

According to BIC database [10] the majority of the pathogenic mutations in *BRCA1/2* are frameshift mutations (around 70 %), while nonsense and missense mutations contribute with around 10 % each. In the present study we have identified only two previously reported *BRCA1* point mutations with clear pathogeniceffect:c.181T > G and c.4603G > T, located in exon 5 and 15, respectively (Table 2). The mutation c.4603G > T was reported several times in BIC as clinically important and it was found in Non-AJ patients from America and Venezuela [10, 39]. Among the studied Bulgarian patients c.4603G > T was observed only once (0.5 %) and was associated with early onset BC (BC37, Table 3).

Following the most prevalent mutation c.5263_5264insC, the missense variant c.181T > G has been recognised as the second most frequent mutation in Poland and other European countries among BC/OC families [13]. These two founder mutations accounted for 70–90 % of the *BRCA1* mutations found in the Polish population, 80 % in Hungary and 28 % in Germany, respectively [13]. Located in the RING domain of *BRCA1*, c.181T > G has been found to impede the coordination of the zinc ions upon binding to the protein in functional studies [25].

In Austria, Slovenia, and Czech Republic the families with c.181T > G mutation represented 15 %, 18 % and 9 %, respectively, of all families carrying *BRCA1* mutations [13]. It has been also known as one of the three founder mutations in Byelorussian population with frequencies of 1 % in unselected BC families and 0.2 % in control individuals [40]. The mutation was detected at a very low rate in our neighboring countries such as Greece, Serbia and Romania, as well as in Croatia, Austria, Slovakia, Ukraine, Latvia, Lithuania and Russia [13]. In our studyc.181T > G was found as a disease causing mutation in two patients (1 %), with early onset (BC132) and BBC (BC28), respectively (Table 3).

We have identified two new frameshift mutations in the *BRCA1* gene: c.464delA and c.5397_5403delCCCTTGG with 0.5 % frequency each (Table 2). The mutation c.464delA, located in exons 8, leads to a truncated protein, lacking its Coiled Coil and BRCT domains and was detected in a patient BC134 with TNBC diagnosed at the age of 54 with three cases of BC in her pedigree (Table 3). Three of her healthy first-degree relatives (sister, and 2 sons) were also carriers of the mutation (Additional file 3: Figure S1). The second novel mutation c.5397_5403delCCCTTGG in exon 22 residing in the BRCT motif leads to a truncated protein without C-terminal BRCA2/AD2 domain. It was found in an individual BC205 with TNBC diagnosed at the age of 51 with family history of BC (Table 3, Additional file 4: Figure S2).

### BRCA2 mutations

The second in frequency (2 %) mutation observed in our study was the insertion c.9098_9099insA, located in exon 23 of *BRCA2* gene. It has been reported as one of the most frequent *BRCA1/2* mutations in Germany with a possible founder effect [13]. Together with c.5946delT it also accounted for 50 % of all *BRCA2* mutations in BC/OC families from Hungary [13]. The consequence of this mutation is a premature stop codon at position 3042 that leads to a truncated protein losing its C terminal OB3 and NLC motifs and thus the ability to bind a DSS1 protein that regulates its repairing function (Table 2). All four carriers of the insertion c.9098_9099insA in our study (BC32, BC81, BC85 and BC88) had family history of BC, and were diagnosed with BC between 50 and 61 years of age. One of them (BC88) developed TNBC (Table 4).

Three of the observed known frameshift mutations were located in exon 11 of *BRCA2* (Table 2): c.5851_5854delAGTT, c.5946delT, and c.5718_5719delCT. The deletion c.5851_5854delAGTT has been detected with a low frequency in cohorts of Italian, American Asian and Indian BC/OC patients [41, 42]. In the present investigation we have identified two (1 %) unrelated Bulgarian patients harbouring c.5851_5854delAGTT deletion. One of the patients (BC76) with a family history of BC and stomach cancer was diagnosed with TNBC at the age of 53, while the other (BC58) developed BC at the age of 48 and had a family history of BC and CRC (Table 4).

The c.5946delTwas found in one patient with Jewish origin and family history of BC (BC52), who had developed both BBC at the age of 41/68 and OC at the age of 59 (Table 4). A healthy daughter of the patient also harboured the mutation. The c.5946delT deletion has been recognised as one of the three founder mutations in AJ, together with the two *BRCA1* indels c.68_69 delAG and c.5263_5264insC [13]. The mutation c.5946delT has been also detected in non-Jewish populations such as the Hungarian BC/ OC families [13].

The third mutation, observed in exon 11 of *BRCA2* c.5718_5719delCT, was reported several times in BIC database [10]. It has been found with a very low frequency in patients with BC, OC and PC from Germany, UK and North America [10, 43]. The mutation was found with similar low frequency (0.5 %) in our study and correlated with early onset (BC90, Table 4).

Another recurrent mutation according to BIC database [10], c.7910_7914delCCTTT, located in *BRCA2*exon 17, was identified in a proband BC19 (0.5 %) with early onset BBC (at the age of 37/41) with family history of BC/OC (Table 4). A cousin of the patient also harboured the mutation and possessed identical clinical phenotype. Interestingly, c.7910_7914delCCTTT has been associated with BBC in other European populations as well [40]. It was first reported in UK and was further observed with low frequencies in patients with BBC from Germany and Denmark and with a frequency of 3.6 % (1/27) among males with sporadic BC in the Polish population [44, 45].

Two novel frameshift mutations were found in *BRCA2*: c.8532_8533delAA and c.9682delA with frequency of 0.5 % each (Table 2). The mutation c.8532_8533delAA, located in exon 20, was found in a patient BC87 with BBC (at the age of 30 and 37) and early onset (Table 4). The patient’s mother was also diagnosed with BBC and carried the mutation (Additional file 5: Figure S3). This mutation is located in the Tower domain of the protein and terminates the translation at the middle of OB2 domain as a result of which BRCA2 lacks its OB3 and NLC motifs at the C terminus. Similarly the second novel *BRCA2* deletion located in exon 27 was observed in a patient BC24 with BBC (Table 4). The consequence of this mutation is a truncated protein without NLC2 and 3 motifs at the C-terminus.

### Unclassified variants

In addition to the unequivocally damaging mutations, many VUSs have been reported in index cases of high-risk BC/OC families in the absence of pathogenic mutation, and their effect on the protein structure and function could not be immediately inferred [46]. Classifying these variants of unknown clinical significance as neutral or disease causing is very important for the genetic counseling.

We have identified in total 50 VUSs in both genes: 24 in *BRCA1* and 26 in *BRCA2*, of which 24 were missense variants (Table 5). The *in silico* analysis suggested a possible deleterious effect of only five missense variants with all prediction programs (c.1067A > G, c.1648A > C and c.3113A > G in *BRCA1* and 3515C > T and c.6100C > T in *BRCA2*) (Table 6). Another two: 139 T > G and c.536A > G in *BRCA1* appeared to be clinically important according to POLYPHEN 2 and SIFT only (Table 6). One new *BRCA1* missense variant c.3999 T > C was observed in a patient BC20 with BBC and early onset in the absence of other pathogenic mutations, but the *in silico* analysis did not predict a possible pathogenic effect (Table 6).

The variant c.139 T > G that appeared to be clinically important upon *in silico* analysis is colocalized with the missense mutation c.181T > G in the RING finger domain of *BRCA1* (exon 5) and has been recognized as one of the zinc-coordinating residues of the protein [10]. It was reported once in BIC database as a VUS [10]. Up to date there is no information available about its allele frequencies in different populations. Several functional studies have suggested a pathogenic effect of c.139T > G since in cell culture experiments it caused disability in coordination of the zinc ions in the RING domain and abolished the ubiquitin ligase activity of *BRCA1* protein as well as its participation in the homologous recombination and the control of the centrosome number [25]. In our study the missense variantc.139T > G was found in one patient (0.5 %) with TNBC and early onset of the disease BC10, in the absence of other pathogenic mutation. However in order to ascertain its pathogenicity, further analysis of large genomic rearrangements in *BRCA1/2* genes, as well as segregation analysis in the family of the patient need to be performed.

For all other missense variants that were predicted pathogenic by the *in silico* analysis *(*Table 6), the existing information in the literature has been controversial. For example, in some studies no association with BC has been found for the *BRCA1* VUS c.1067A > G (Q356R), although the authors claimed that being homozygous for 356R might protect against BC [46]. According to other prediction models it demonstrated a possible harmful role [46]. Similar results have been obtained for the c.536A > G, c.1648A > C, c.3113A > G in *BRCA1* and 3515C > Tin *BRCA2* [46]. Functional studies suggested that c.536A > G but not c.1648A > C variant might be related to *BRCA1*-associated pathogenicity by affecting its function in nonhomologous end joining (NHEJ) [25].

In the present study c.536A > G and c.1648A > C in *BRCA1*, as well as c.3515C > T in *BRCA2* (Table 5) were observed only in patients, with a low frequency (MAF < 0.005). In contrast 1067A > G and c.3113A > G in *BRCA1* (Table 5) were detected in both patients and controls with a high frequency (MAF > 0.005).

The c.536A > G, c.1456 T > C and c.1648A > C have been often seen together and probably constitute a rare haplotype [47]. One of these substitutions, c.1456T > C was classified as neutral in the *in silico* analysis, in contrast to the other two (Table 6). We have observed this haplotype in one patient BC117 with family history of BC diagnosed at the age of 48. Her sister also developed BC at the age of 42, and subsequently died of the disease. We genotyped the patient’s healthy daughter and the two healthy daughters of her sister. One of the proband’s nieces was also carrier of the three missense variants. Identical case was reported in a Sicilian family where the proband with early onset of BC, her mother with early onset of uterine cancer and her healthy sister harboured the haplotype 536A > G, c.1456T > C, c.1648A > C [47]. It has been suggested that the three amino acid changes might alter the charge and stoichiometry of the protein and in consequence its function [47].

Even though the *in silico* analyses might assign deleterious function to some VUSs, they are not always consistent with the biological evidences. Further functional assays are necessary to prove the pathogenic effect of all missense variants predicted to be deleterious in current and previous studies.

## Conclusions

As a result of the present study a mutation profile of the *BRCA1*/*2* genes in Bulgarian BC/OC patients has been established with 13 (11 frameshift, 1 nonsense, 1 missense) unequivocally disease causing mutations**.** Mutations in *BRCA1* gene were found in 14 % (28/200) and in *BRCA2* in 5.5 % (11/200) of the Bulgarian patients selected by the recognized criteria for *BRCA1/2* genetic testing. Four new frameshift (2 in *BRCA1* and 2 in *BRCA2* genes) have been found. Altogether inherited BC predisposition was identified in 39 (19.5 %) of the patients.

The most prevalent mutation in Eastern Europe c.5263_5264insC appeared to have a founder effect in the Bulgarian population with an overall frequency of 11 % in the studied cohort of familial BC/OC patients. It was also found in 14 % of the patients with TNBC without family history. Together with the other 3 recurrent mutations identified (c.181T > G in *BRCA1*; c.9098_9099insA and c.5851_5854delAGTT in *BRCA2*) they account for 77 % of all detected mutations. However, MLPA analysis is necessary to be performed in order to ascertain the contribution of the large genomic indels and rearrangements in *BRCA1/2* genes for familial BC/OC development in the Bulgarian population.

Consequently, we suggest a mutation screening pipeline in which an initial test is performed to specifically detect *BRCA1* c.5263_5264insC and the 3 additional recurrent mutations in BC patients from severely affected families to identify about two thirds of the carriers. In the remaining patients without mutations, complete sequencing of the coding regions of *BRCA1* and *BRCA2* is warranted, followed by MLPA screening. Such an approach would improve the effectiveness of the protocol and reduce the costs of mutation screening. This may have direct effect on the efficient molecular diagnostics of the genetic predisposition to BC/OC in Bulgaria.

### Availability of supporting data

The data sets supporting the results of this article are included within the article and its additional files. The distribution of patients by criteria is represented in Additional file 1: Table S1. The list of primers sequences used for PCR amplification of the entire exons and exon-intron junctions of the *BRCA1* and *BRCA2* genes is shown in Additional file 2: Table S2. The pedigrees of the patients harboring the novel damaging mutations c.464delA and 5397_5403delCCCTTGG in *BRCA1*, and c.8532_8533delAA in *BRCA2* are represented in Additional file 3: Figure S1, Additional file 4: Figure S2 and Additional file 5: Figure S3, respectively.

## Additional files

Additional file 1: Table S1. Total number of BC patients distributed by criteria.
Additional file 2: Table S2. List of primers sequences used for PCR amplification of the entire exons and exon-intron junctions of the *BRCA1* and *BRCA2* genes.
Additional file 3: Figure S1. A pedigree of the patient BC134 harboring the novel damaging mutation c.464delA in *BRCA1*. The patient was diagnosed with TNBC at the age of 54 with three cases of BC in her family. Three of her healthy first degree relatives (sister, and 2 sons) were also carriers of the mutation.
Additional file 4: Figure S2. A pedigree of the breast cancer (BC) patient BC205 harboring the novel damaging mutation c.5397_5403delCCCTTGG in *BRCA1*. The patient was diagnosed with TNBC at the age of 51 and had a personal history of brain papilloma (BP) at the age of 18 and thyroid adenoma (TA) around 30 years of age. Her mother and great-grandmother were also diagnosed with BC at the age of 51 and 30, respectively. The healthy (Ht) sister was screened for the presence of the mutation and was not a carrier (wt –wild type).
Additional file 5: Figure S3. A pedigree of the patient BC87 harboring the novel damaging mutation c.8532_8533delAA in *BRCA2*. The patient was diagnosed with bilateral breast cancer (BBC) and had early onset (y 30; y 37) of the disease. Similarly her mother was diagnosed with early onset BBC (y 37; y 38) and also carried the mutation.
