# Erratum to: Spectrum and frequencies of BRCA1/2 mutations in Bulgarian high risk breast cancer patients

**DOI:** 10.1186/s12885-017-3188-6

**Published:** 2017-03-16

**Authors:** Rumyana Ivanova Dodova, Atanaska Velichkova Mitkova, Daniela Rosenova Dacheva, Lina Basam Hadjo, Alexandrina Ivanova Vlahova, Margarita Stoyanova Taushanova – Hadjieva, Spartak Stoyanov Valev, Marija Mitko Caulevska, Stanislava Dimitrova Popova, Ivan Emilov Popov, Tihomir Iliichev Dikov, Theophil Angelov Sedloev, Atanas Stefanov Ionkov, Konstanta Velinova Timcheva, Svetlana Liubomirova Christova, Ivo Marinov Kremensky, Vanio Ivanov Mitev, Radka Petrova Kaneva

**Affiliations:** 1 Molecular Medicine Center, Medical University of Sofia, 2 Zdrave str, 1431 Sofia, Bulgaria; 2 Department of Medical Chemistry and Biochemistry, Medical Faculty, Medical University of Sofia, 2 Zdrave str, 1431 Sofia, Bulgaria; 3 General and Clinical Pathology Clinic, University Hospital “Alexandrovska”, 1 Georgi Sofiiski str, 1431 Sofia, Bulgaria; 4 Department of General and Clinical Pathology, Medical University of Sofia, 1 Georgi Sofiiski str, 1431 Sofia, Bulgaria; 5 Clinic of Medical Oncology (Chemotherapy), Specialized Hospital for Active Treatment in Oncology, 6 “Plovdivsko pole” str, 1756 Sofia, Bulgaria; 6 Department of Surgery, University Hospital “Tsaritsa Yoana - ISUL”, 8 “Byalo more” str, 1527 Sofia, Bulgaria; 7 Medical Faculty, 8 “Byalo more” str, 1527 Sofia, Bulgaria; 8 Department of General and Liver-Pancreatic Surgery, University Hospital “Alexandrovska”, 1 Georgi Sofiiski str, 1431 Sofia, Bulgaria; 9 Medical Faculty, Medical University of Sofia, 1 Georgi Sofiiski str, 1431 Sofia, Bulgaria

## Erratum

In this version of this article that was originally published [1] there was an error with the citation in the line: “…Functional studies suggested that c.536A > G but not c.1648A > C variant might be related to BRCA1-associated pathogenicity by affecting its function in non-homologous end joining (NHEJ) [25]…”

The reference number 25 is incorrect for this citation. The correct reference does not appear in the article, but should be:

Guidugli L, Rugani C, Lombardi G, Aretini P, Galli A, Caligo MA. A recombination-based method to characterize human BRCA1 missense variants. Breast Cancer Red Treat. 2011 125(1):265-72.

## References

[1] Dodova RI, Mitkova AV, Dacheva DR, Hadjo LB, Vlahova AI, Taushanova-Hadjieva MS (2015). **Spectrum and frequencies of BRCA1/2 mutations in Bulgarian high risk breast cancer patients**. BMC Cancer..

