# Supplementary material for: Spectrum and frequencies of BRCA1/2 mutations in Bulgarian high risk breast cancer patients
Source: BMC Cancer. 2015 Jul 17;15:523. doi: 10.1186/s12885-015-1516-2 (PMC4504066; doi:10.1186/s12885-015-1516-2)
Supplement: Additional file 1: Table S1. — Total number of BC patients distributed by criteria. [file 12885_2015_1516_MOESM1_ESM.pdf]

Additional Table 1. Total number of BC patients distributed by criteria

Criteria	Number of BC patients
BCLC-1. An affected individual with BC with two first-degree relatives (mother, daughter or sister) of which at least one is diagnosed with BC under the age of 40 or OC at any age.	6
BCLC-2. An affected individual with BC with three or more first-degree or second-degree (grandmother or aunt) relatives diagnosed with BC or OC regardless of their age of diagnosis.	31
BCLC-3. An affected individual with early-age-onset BC diagnosed under the age of 40.	55
BCLC-4. An affected individual with BBC.	30
NCCN-1. An affected individual with two or more clearly separate ipsilateral primary tumours, or breast and ovarian/ fallopian tube/ primary peritoneal cancer.	5
NCCN-2. An affected individual with family history of two or more breast primaries including bilateral disease or two or more clearly separate ipsilateral primary tumours, as well as breast and ovarian/ fallopian tube/ primary peritoneal cancers in close relative(s) from the same side of the family (maternal or paternal).	38
NCCN-3. An affected individual with family history of BC in combination with one or more of the following cancers: thyroid cancer, sarcoma, adrenocortical carcinoma, endometrial cancer, pancreatic cancer, brain tumours, diffuse gastric cancer, dermatological manifestations of the Cowden's syndrome or leukemia/ lymphoma on the same side of the family (maternal or paternal).	7
TNBC. An affected individual with TNBC.	28
Total number of patients	200
