# Supplementary material for: Spectrum and frequencies of BRCA1/2 mutations in Bulgarian high risk breast cancer patients
Source: BMC Cancer. 2015 Jul 17;15:523. doi: 10.1186/s12885-015-1516-2 (PMC4504066; doi:10.1186/s12885-015-1516-2)
Supplement: Additional file 2: Table S2. — List of primers sequences used for PCR amplification of the entire exons and exon-intron junctions of the BRCA1 and BRCA2 genes. [file 12885_2015_1516_MOESM2_ESM.pdf]

Additional Table 2. List of primers sequences used for PCR amplification of the entire exons and exon-intron junctions of the *BRCA1* and *BRCA2* genes

Exon	F/R	Nucleotide sequence of the primers	Size of PCR products	Annealing temperature	Design by
		BRCA1			
2	F	5′-GGACGTTGTCATTAGTTCTTTGG-3′	339bp	56 ºC	custom
	R	5′-GTCTTTTCTTCCCTAGTATGT-3′			bic
3	F	5′-AACGAACTTGAGGCCTTATG-3′	308bp	51ºC	bic
	R	5′-TTGGATTTTCGTTCTCACTT-3′			bic
5	F	5′-CTCTTAAGGGCAGTTGTGAG-3′	278bp	56 ºC	bic
	R	5′-ATGGTTTTATAGGAACGCTATG-3′			bic
6	F	5′-TTTCTACTGTTGCTGCATCT-3′	273bp	58 ºC	custom
	R	5′-TAATGTGCAAACTTCCTGAG-3′			custom
7	F	5′-GGTTTCTCTTGGTTTCTTTG-3′	250bp	57 ºC	bic
	R	5′-CCCAGCTACTAAGGGGGCTA-3′			custom
8	F	5′-TGTTAGCTGACTGATGATGGT-3′	268bp	54 ºC	bic
	R	5′-AACCCAGCAATTATTATTAAATAC-3′			bic
9	F	5′-CCACAGTAGATGCTCAGTAAATA-3′	211bp	57 ºC	bic
	R	5′-TAGGAAAATACCAGCTTCATAGA-3′			bic
10	F	5′-GATCTTGGTCATTTGACAGTTC-3′	240bp	53 ºC	bic
	R	5′-CCCAAATGGTCTTCAGAATA-3′			bic
11a	F	5′-CCACCTCCAAGGTGTATGA-3′	370bp	59 ºC	custom
	R	5′-TGTTATGTTGGCTCCTTGCT-3′			custom
11b	F	5′-TTACTCACTAAAGACAGAATGA-3′	401bp	58 ºC	custom
	R	5′-CCAGAATATTCATCTACCTCA-3′			custom
11c	F	5′-CACATGATGGGGAGTCTGA-3′	366bp	58 ºC	custom
	R	5′-TCTTGATAAAATCCTCAGGATG-3′			custom
11d	F	5′-CAAGAGCGTCCCCTCACA-3′	388bp	63 ºC	custom
	R	5′-GCGCATGAATATGCCTGGT-3′			custom
11e	F	5′-GTATAAGCAATATGGAACTCGA-3′	388bp	59 ºC	custom
	R	5′-TTAAGTTCACTGGTATTTGAACA-3′			custom
11f	F	5′-GACAGCGATACTTTCCCAGA-3′	704bp	60 ºC	custom
	R	5′-TGGAACAACCATGGATTAGTC-3′			custom
11g	F	5′-GGAAGTTAGCACTCTAGGGA-3′	423bp	57 ºC	custom
	R	5′-GCAGTGATATTAACTGTCTGTA-3′			custom
11h	F	5′-GCAACATTCTCTGCCCACTC-3′	436bp	58 ºC	custom
	R	5′-GGCTAATTGTGCTCACTGT-3′			custom
11i	F	5′-CTACTTTTTCCCATCAAGTCA-3′	377bp	59 ºC	custom
	R	5′-TCAGGATGCTTACAATTACTTC-3′			custom
11j	F	5′-CAAAATTGAATGCTATGCTTAGA-3′	377bp	61 ºC	custom
	R	5′-TCGGTAACCCTGAGCCAAAT-3′			custom
11k	F	5′-GCAAAAGCGTCCAGAAAGGA-3′	396bp	61 ºC	custom
	R	5′-TATTTGCAGTCAAGTCTTCCAA-3′			custom
11l	F	5′-GTAATATTGGCAAAGGCATCT-3′	360bp	63 ºC	custom
	R	5′-TAAAATGTGCTCCCCAAAAGCA-3′			custom
12	F	5′-GCGTTTATAGTCTGCTTTTACA-3′	227bp	54 ºC	bic
	R	5′-TGTCAGCAAACCTAAGAATGT-3′			bic
13	F	5′-AATGGAAAGCTTCTCAAAGTA-3′	320bp	54 ºC	bic
	R	5′-ATGTTGGAGCTAGGTCCTTAC-3′			bic
14	F	5′-CTAACCTGAATTATCACTATCA-3′	312bp	54 ºC	bic
	R	5′-GTGTATAAATGCCTGTATGCA-3′			bic
15	F	5′-CAGACTTCTAGGCTGTCTTGC-3′	378bp	59 ºC	bic
	R	5′-GTGTTTGTTCCAATACAGCAG-3′			bic
16	F	5′-AATTCTTAACAGAGACCAGAAC-3′	450bp	56 ºC	bic
	R	5′-AAAACTCTTTCCAGAATGTTGT-3′			bic
17	F	5′-AGCTGTGTGCTAGAGGTAACTC-3′	190bp	60 ºC	bic
	R	5′-GTGGTTTTATGCAGCAGATG-3′			bic
18	F	5′-AGCCTCCCCTAGACTTCCAA-3′	319bp	60 ºC	custom
	R	5′-TAAAGGGAGGAGGGGAGAAA-3′			custom
19	F	5′-TGTGGCTTTAGAGGGAAGGA-3′	261bp	60 ºC	custom
	R	5′-GGTGCATTGATGGAAGGAAG-3′			custom
20	F	5′-ATATGACGTGTCTGCTCCAC-3′	259bp	54 ºC	bic
	R	5′-AGTCTTACAAAATGAAGCGG-3′			bic

	F	5′-AAGCTCTTCCTTTTTGAAAGTC-3′		58 ºC	bic
21	R	5′-GTAGAGAAATAGAATAGCCTCT-3′	298bp	58 ºC	bic
	F	5′-TCCCATTGAGAGGTCTTGCT-3′			bic
	R	5′-GAGAAGACTTCTGAGGCTAC-3′			bic
23	F	5′-TGAAGTGACAGTTCCAGTAGT-3′	258bp	58 ºC	custom
	R	5′-CATTTTAGCCATTCATTCAACAA-3′			custom
24	F	5′-ATGAATTGACACTAATCTCTGC-3′	280bp	58 ºC	bic
	R	5′-GTAGCCAGGACAGTAGAAGGA-3′			bic
		BRCA2			
2	F	5′-CCAGGAGATGGGACTGAATTAG-3′	311bp	60 ºC	bic
	R	5′-CTGTGACGTACTGGGTTTTTAGC-3′			bic
3	F	5′-GATCTTTAACTGTTCTGGGTCACA-3′	424bp	59 ºC	bic
	R	5′-CCCAGCATGACACAATTAATGA-3′			bic
4	F	5′-AAACACTTCCAAAGAATGCAAA-3′	399bp	54 ºC	custom
	R	5′-CCAGCCAATTCAACATCACA-3′			custom
5/6	F	5′-TGTGTTGGCATTTTAAACATCA-3′	453bp	54 ºC	bic
	R	5′-CAGGGCAAAGGTATAACGCT-3′			bic
7	F	5′-CCTTAATGATCAGGGCATTTC-3′	215bp	59 ºC	bic
	R	5′-CAACCTCATCTGCTCTTTCTTG-3′			bic
8	F	5′-GCCATATCTTACCACCTTGTGA-3′	406bp	60 ºC	bic
	R	5′-AGGTTTAGAGACTTTCTCAAAGGC-3′			bic
9	F	5′-ATAACTGAAATCACCAAAAGTG-3′	243bp	62 ºC	bic
	R	5′-CTGTAGTTCAACTAAACAGAGG-3′			bic
10a	F	5′-CAGGAGAAGGGGTGACTGAC-3′	575bp	61 ºC	custom
	R	5′-AGACCATTCACAGGCCAAAG-3′			custom
10b	F	5′-ATTTTCCATGAAGCAAACGC-3′	720bp	57 ºC	custom
	R	5′-AGAATTCTGTGTGGTGGTGG-3′			custom
10c	F	5′-TGGAGAAAATACCCCTATTGC-3′	491bp	58 ºC	custom
	R	5′-AGAATTCTGTGTGGTGGTGG-3′			custom
10d	F	5′-TCACCTAAAGAGACTTTCAATGC-3′	442bp	61 ºC	custom
	R	5′-CAGAAGGAATCGTCATCTATAAAAC-3′			custom
11a	F	5′-CACTGTGCCCAAACACTACC-3′	462bp	56 ºC	custom
	R	5′-CTTTTCTGGGATTGAAAGTCAG-3′			custom
11b	F	5′-CCTGCAGGAAGGACAGTGTG-3′	410bp	61 ºC	custom
	R	5′-TTCTTGAAGGTGATGCTACTCTC-3′			custom
11c	F	5′-CCCATGGAAAAGAATCAAGATG-3′	390bp	61 ºC	custom
	R	5′-AATTGACACTTGGGTTGCTTG-3′			custom
11d	F	5′-CGAACCCATTTTCAAGAACTC-3′	432bp	58 ºC	custom
	R	5′-GGCTTGCTCAGTTTCTTTTGA-3′			custom
11e	F	5′-CAATTTCAAATCACAGTTTTGG-3′	452bp	56 ºC	custom
	R	5′-CATCTGGTTTTCAGGCACTTC-3′			custom
11f	F	5′-AACACCTAGCCAAAAGGCAG-3′	396bp	56 ºC	custom
	R	5′-TGCAGAGCTTCAGTAGAAACATTC-3′			custom
11g	F	5′-TTAAACGGAAGTTTGCTGGC-3′	427bp	55 ºC	custom
	R	5′-TTTCTACTGGCAGCAGTATATTTG-3′			custom
11h	F	5′-AAATAATATTGAAATGACTACTGGCAC-3′	451bp	60 ºC	custom
	R	5′-TTTATTAAATGACTCTTTGGCGAC-3′			custom
11i	F	5′-TTTTTGGAAGTTGCGAAAGC-3′	455bp	54 ºC	custom
	R	5′-TCCAAAGATTCCTTTGCAATTT-3′			custom
11j	F	5′-TGAAAGAAAGTGTCCCAGTTG-3′	419bp	55 ºC	custom
	R	5′-TGATGTTTTGAGATTTTCAGTTTG-3′			custom
11k	F	5′-AGCTGCCCCAAAGTGTAAAG-3′	506bp	59 ºC	custom
	R	5′-CATCAGAATGGTAGGAATAGCTG-3′			custom
11l	F	5′-TCATTGAAAATTCAGCCTTAGC-3′	458bp	55 ºC	custom
	R	5′-AGTCACAAGTTCCTCAACGC-3′			custom
11m	F	5′-ATGCAAATGCATACCCACAA-3′	442bp	56 ºC	custom
	R	5′-GAAACTTTCTCCAATCCAGACA-3′			custom
11n	F	5′-CAAAAATTTGCCAAACGAAA-3′	432bp	58 ºC	custom
	R	5′-TGAGCTGGTCTGAATGTTCG-3′			custom
11o	F	5′-TCATCTGCAAATACTTGTGGG-3′	440bp	54ºC	custom
	R	5′-TGCTCTGGGTTTCTCTTATCAAC-3′			custom
11p	F	5′-CAAGTTTCCATTTTAGAAAGTTCC-3′	408bp	56 ºC	custom
	R	5′-AAAAGTTTCAGTTTTACCAATTTCC-3′			custom
11q	F	5′-TCAATTTCAACAAGACAAACAACA-3′	521bp	62 ºC	custom
	R	5′-TCAAACCATACTCCCCCAAA-3′			custom
12	F	5′-AGTGGTGTTTTAAAGTGGTCAAAA-3′	388bp	55 ºC	bic
	R	5′-GGATCCACCTGAGGTCAGAATA-3′			bic

13	F	5′-TGCTGATTTCTGTTGTATGCTTG-3′	546bp	61 ºC	custom
	R	5′-TCCTCTCAACCTTAGTACTTCATCC-3′			custom
14	F	5′-ACCATGTAGCAAATGAGGGTCT-3′	557bp	62 ºC	bic
	R	5′-GCAAAAAATTCATCACACAAATTGTC-3′			bic
15	F	5′-GGCCAGGGGTTGTGCTTTTT-3′	314bp	54 ºC	bic
	R	5′-AGGATACTAGTTAATGAAATA-3′			bic
16	F	5′-TTTGGTAAATTCAGTTTTGGTTTG-3′	350bp	59 ºC	bic
	R	5′-GAGAAGAAAGAGGGATGAGGG-3′			custom
17	F	5′-CACCATGCTCAGCAATGAAG-3′	485bp	57 ºC	custom
	R	5′-CACTGACAACTGGCTTGTGC-3′			custom
18a	F	5′-TGATCCACTATTTGGGGATTG-3′	466bp	56 ºC	custom
	R	5′-GAGGGGAGGATCTAACTGGG-3′			custom
18b	F	5′-ATGGAAAGGGATGACACAGC-3′	502bp	62 ºC	custom
	R	5′-CTCAAGAAAGATCTCTGGACCTC-3′			custom
19	F	5′-AAGTGAATATTTTTAAGGCAGTT-3′	296bp	56 ºC	bic
	R	5′-TATATGGTAAGTTTCAAGAAT-3′			bic
20	F	5′-CAAGTAGCTGAGCCACCACA-3′	623bp	56 ºC	custom
	R	5′-ACAAAAAATGCAAAAGCCACA-3′			custom
21	F	5′-TGCTTGGTTCTTTAGTTTTAGTTGC-3′	261bp	60 ºC	custom
	R	5′-GAGTCTAAAACAGCTTCTCACCTTG-3′			custom
22	F	5′-AACCACACCCTTAAGATGAGC-3′	455bp	61 ºC	bic
	R	5′-GGGCATTAGTAGTGGATTTTGC-3′			bic
23	F	5′-GCAAAATCCACTACTAATGCCC-3′	350bp	57 ºC	custom
	R	5′-TGGAGATTCCATAAACTAACAAGC-3′			custom
24	F	5′-CCGGTACAAACCTTTCATTG-3′	329bp	58 ºC	custom
	R	5′-TGCCAACTGGTAGCTCCAAC-3′			custom
25	F	5′-TGCATCTTAAAATTCATCTAACACATC-3′	383bp	62 ºC	custom
	R	5′-AAGCTATTTCCTTGATACTGGACTG-3′			custom
26	F	5′-TGACAATTGGTATCACATTTAGGG-3′	421bp	59 ºC	custom
	R	5′-CAGGAGCCACATAACAACCA-3′			custom
27a	F	5′-TAGGGGAGGGAGACTGTGTG-3′	383bp	59 ºC	custom
	R	5′-ACTCCTTGGTGGCTGAAATG-3′			custom
27b	F	5′-GAAAGAGATTGATGACCAAAAGAA-3′	401bp	61 ºC	custom
	R	5′-GAGATGTAGTACAACGTCGTTTCAG-3′			custom
27c	F	5′-CTGAATTCTCCTCAGATGACTCC-3′	447bp	61 ºC	custom
	R	5′-AAACGCTGAGGTAAATTTGAAAC-3′			custom
27d	F	5′-GGCCAGTACGGAAGAATGTG-3′	556bp	62 ºC	custom
	R	5′-CCTCATTCCATTTAATGACACAA-3′			custom
