# Supplementary figures and images for: Spectrum and frequencies of BRCA1/2 mutations in Bulgarian high risk breast cancer patients
Source: BMC Cancer. 2015 Jul 17;15:523. doi: 10.1186/s12885-015-1516-2 (PMC4504066; doi:10.1186/s12885-015-1516-2)

# BC134

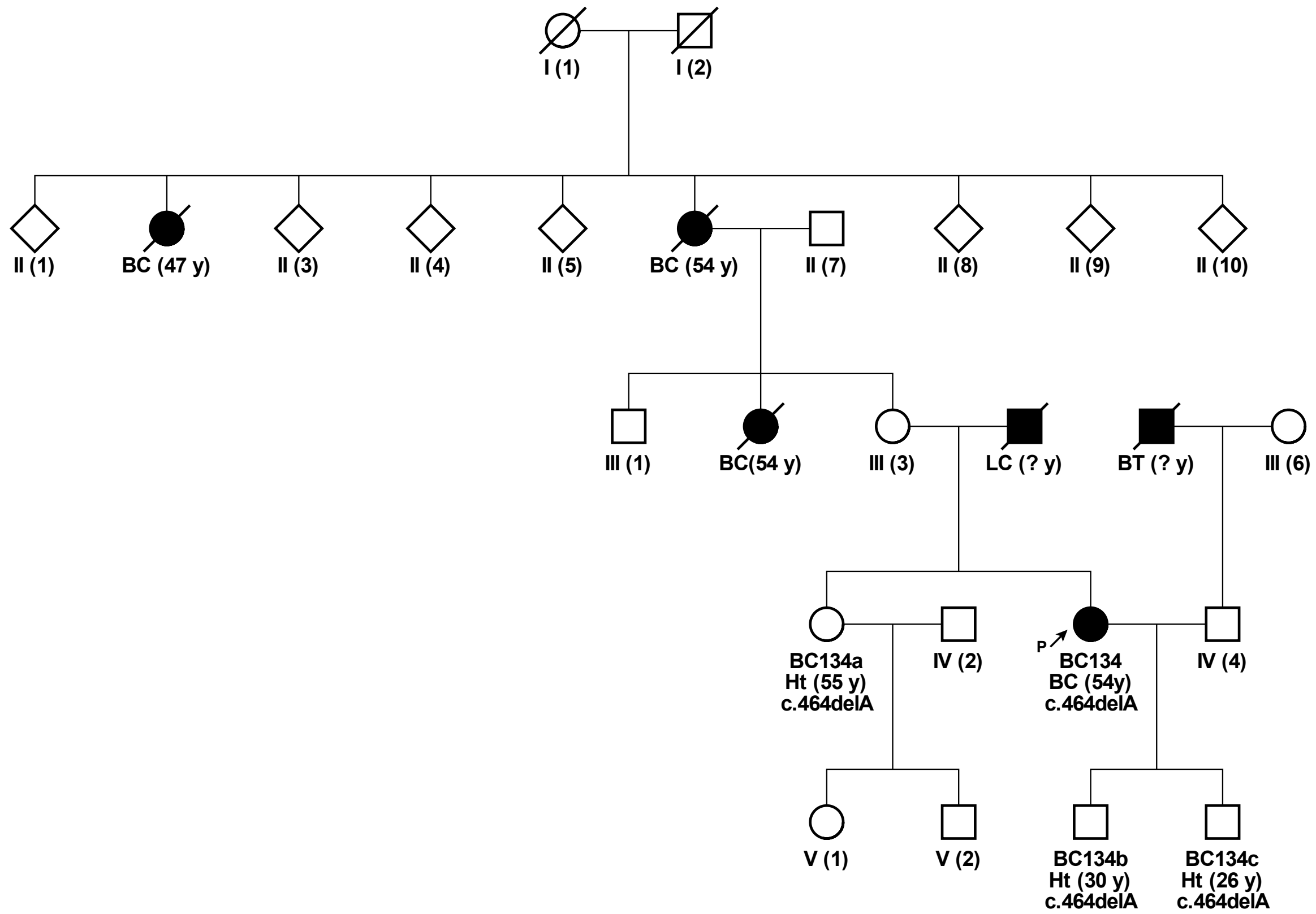

Supplement: Additional file 3: Figure S1. — A pedigree of the patient BC134 harboring the novel damaging mutation c.464delA in BRCA1. The patient was diagnosed with TNBC at the age of 54 with three cases of BC in her family. Three of her healthy first degree relatives (sister, and 2 sons) were also carriers of the mutation. [file 12885_2015_1516_MOESM3_ESM.pdf]

**BC205**

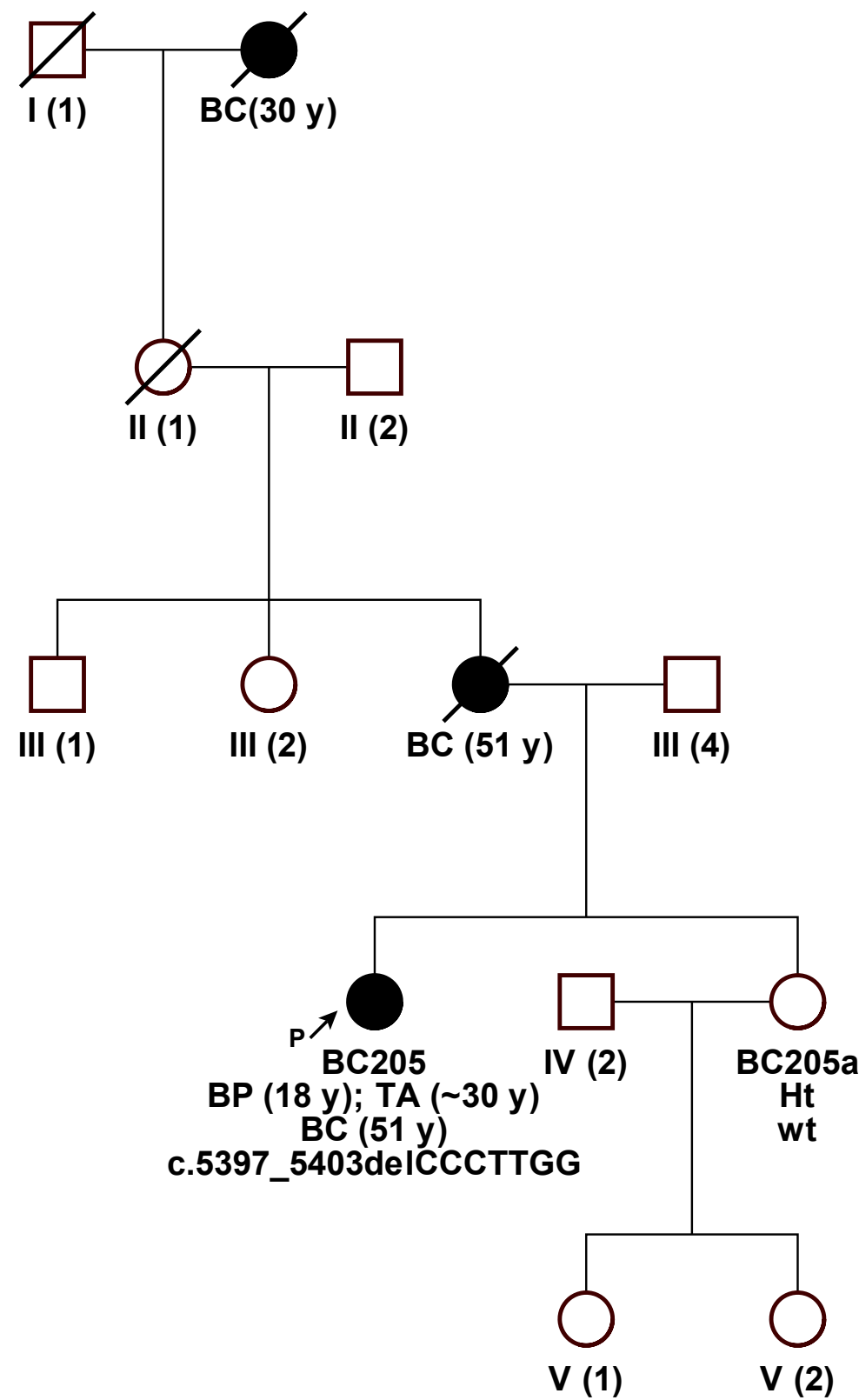

Supplement: Additional file 4: Figure S2. — A pedigree of the breast cancer (BC) patient BC205 harboring the novel damaging mutation c.5397_5403delCCCTTGG in BRCA1. The patient was diagnosed with TNBC at the age of 51 and had a personal history of brain papilloma (BP) at the age of 18 and thyroid adenoma (TA) around 30 years of age. Her mother and great-grandmother were also diagnosed with BC at the age of 51 and 30, respectively. The healthy (Ht) sister was screened for the presence of the mutation and was not a carrier (wt –wild type). [file 12885_2015_1516_MOESM4_ESM.pdf]

# BC087

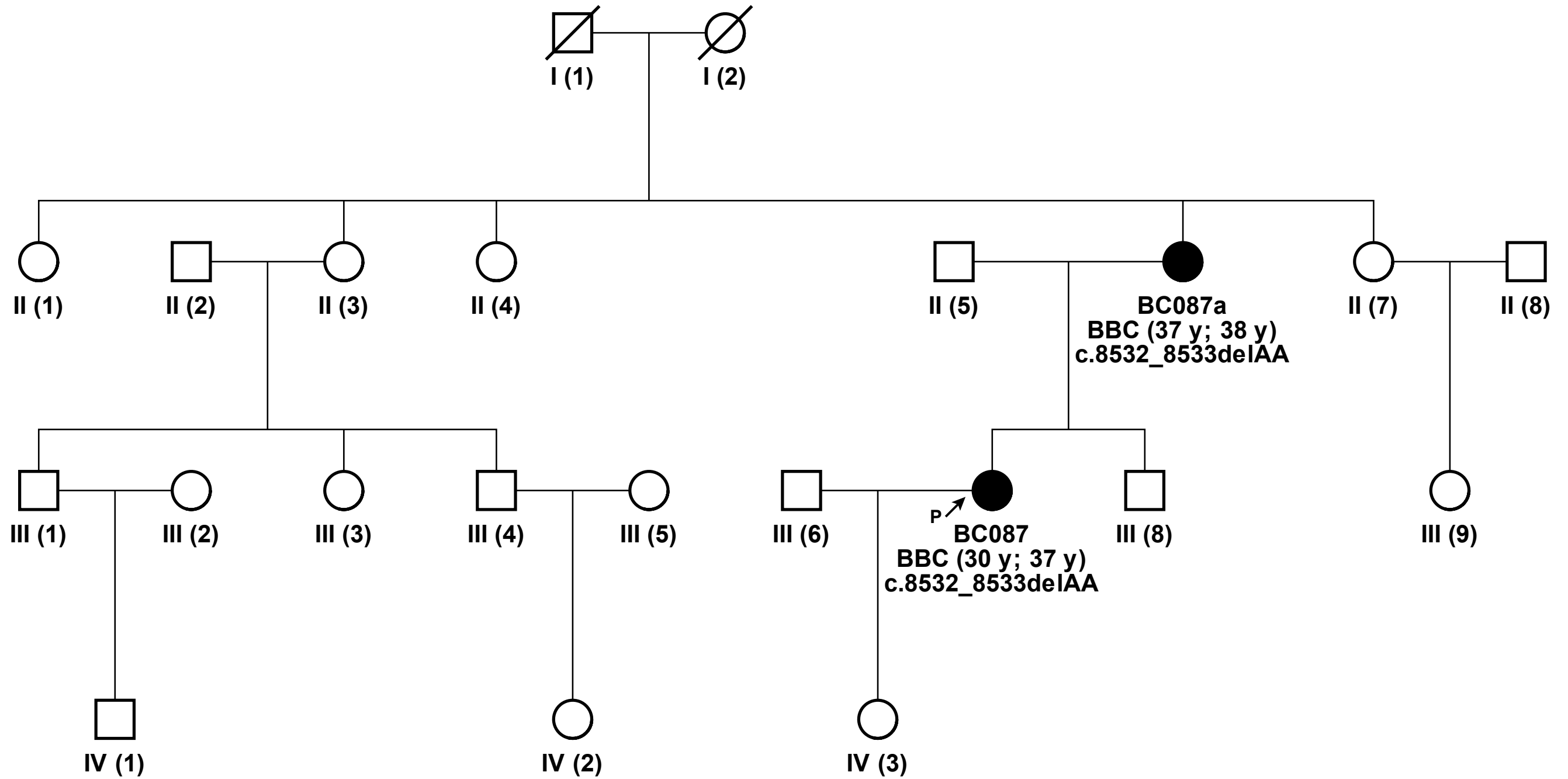

Supplement: Additional file 5: Figure S3. — A pedigree of the patient BC87 harboring the novel damaging mutation c.8532_8533delAA in BRCA2. The patient was diagnosed with bilateral breast cancer (BBC) and had early onset (y 30; y 37) of the disease. Similarly her mother was diagnosed with early onset BBC (y 37; y 38) and also carried the mutation. [file 12885_2015_1516_MOESM5_ESM.pdf]
